# Clinicohistopathological Correlation and Prognostic Significance of Molecular Biomarkers in Urinary Bladder Neoplasms: A Comprehensive Analysis

**DOI:** 10.7759/cureus.66088

**Published:** 2024-08-03

**Authors:** Avinash Mane, Nanda J Patil, Atul B Hulwan, Avishek Koley

**Affiliations:** 1 Department of Pathology, Krishna Institute of Medical Sciences, Krishna Vishwa Vidyapeeth (Deemed to be University), Karad, IND; 2 Department of General Surgery, Sarojini Naidu Medical College, Agra, IND

**Keywords:** neoplasms, urinary bladder, prognostic significance, molecular biomarkers, bladder cancer

## Abstract

Background: Urinary bladder neoplasms constitute a heterogeneous group of tumors with diverse clinical behaviors and outcomes. Understanding the correlation between clinicopathological characteristics and the prognostic significance of molecular biomarkers in bladder cancer is vital for personalized treatment strategies and improved patient outcomes.

Objective: This prospective observational study aimed to comprehensively investigate the clinicopathological correlations and prognostic significance of molecular biomarkers in urinary bladder neoplasms.

Methods: A cohort of 174 patients diagnosed with urinary bladder neoplasm participated in this study. Clinicopathological data, including demographic information, medical history, imaging findings, and histopathological reports, were collected from the patient records. Tissue samples obtained from transurethral resection or biopsy were subjected to molecular biomarker analysis using immunohistochemistry (IHC), fluorescence in situ hybridization (FISH), and molecular profiling techniques. Longitudinal follow-up assessments were conducted to monitor disease progression, recurrence, and overall survival.

Result: Out of 174 patients diagnosed with bladder neoplasms, the mean age of the patients was 62.4 years (±8.7), indicating that the study cohort primarily comprised elderly individuals. The majority of patients were male (126, 72.4%), reflecting the higher prevalence of bladder cancer among men compared to women. Preliminary analysis revealed significant associations between clinicopathological parameters, molecular biomarker expression profiles, and clinical outcomes in patients with urinary bladder neoplasms. Elevated expression levels of specific biomarkers such as tumor protein p53 (p53), Ki-67, and estimated glomerular filtration rate (EGFR) were observed in advanced tumor stages (p < 0.001) and higher histological grades (p < 0.05), indicating their potential prognostic significance. Furthermore, genetic alterations detected using molecular profiling techniques, including chromosomal gains and losses, were significantly correlated with aggressive disease phenotypes and increased recurrence risk (p < 0.01). Longitudinal follow-up data demonstrated that patients with elevated biomarker expression levels or genetic alterations had poorer treatment responses and shorter overall survival durations than those with lower biomarker expression levels.

Conclusion: This study highlights the importance of integrating clinicopathological parameters and molecular biomarker data for the risk stratification, treatment selection, and prognostic assessment of urinary bladder neoplasms.

## Introduction

Urinary bladder neoplasms represent a significant global health concern, comprising a heterogeneous group of tumors with diverse clinical presentations and outcomes [1]. Bladder cancer is the 10th most common cancer worldwide, with an estimated 573,000 new cases diagnosed annually and approximately 212,000 deaths attributed to the disease [2]. Despite advances in diagnostic techniques and treatment modalities, bladder cancer remains a major cause of morbidity and mortality, posing substantial challenges to patients, healthcare providers, and healthcare systems [3].

Bladder cancer is primarily categorized into two broad subtypes based on histological classification: non-muscle-invasive bladder cancer (NMIBC) and muscle-invasive bladder cancer (MIBC) [4]. NMIBC, which includes carcinoma in situ (CIS), Ta, and T1 tumors, accounts for approximately 75% of all bladder cancer cases at initial presentation [5]. Although NMIBC is associated with a favorable prognosis, the risk of disease recurrence and progression to muscle invasion necessitates vigilant surveillance and timely intervention [6]. Conversely, MIBC, characterized by infiltration of the muscularis propria, represents a more aggressive disease phenotype, associated with poorer outcomes and higher mortality rates [7]. The management of MIBC typically involves radical cystectomy with or without neoadjuvant chemotherapy, although bladder-preserving strategies such as trimodal therapy may be considered in select cases [8].

The etiology of bladder cancer is multifactorial, with environmental, occupational, and genetic factors contributing to its pathogenesis. Cigarette smoking is the most well-established risk factor for bladder cancer, accounting for approximately 50% of all cases in industrialized countries [9]. Occupational exposure to carcinogens, such as aromatic amines, polycyclic aromatic hydrocarbons (PAHs), and diesel exhaust, has also been implicated in bladder cancer development, particularly among industrial workers in occupations such as dye manufacturing, rubber processing, and truck driving [10].

In addition to environmental and occupational factors, genetic predisposition plays a significant role in susceptibility to bladder cancer. Several genetic polymorphisms and alterations that modulate an individual’s risk of developing bladder cancer have been identified. For example, single nucleotide polymorphisms (SNPs) in genes encoding enzymes involved in xenobiotic metabolism, DNA repair, and cell cycle regulation have been associated with bladder cancer risk [11]. Moreover, somatic mutations in key oncogenes (e.g., fibroblast growth factor receptor 3 (FGFR3) and phosphatidylinositol-4,5-bisphosphate 3-kinase catalytic subunit alpha (PIK3CA)) and tumor suppressor genes (e.g., tumor protein p53 (TP53) and RB1) have been identified in bladder cancer tumors, driving tumorigenesis and disease progression [12].

Despite the heterogeneity of bladder cancer, there is growing recognition of the importance of molecular biomarkers in the diagnosis, prognosis, and treatment stratification of bladder cancer patients. Molecular biomarkers encompass a wide array of molecular alterations, including genetic mutations, epigenetic modifications, and protein expression profiles, which reflect the underlying molecular pathogenesis of bladder cancer and inform clinical decision-making [13]. These biomarkers have the potential to revolutionize bladder cancer management by enabling personalized treatment approaches, identifying high-risk patients for intensive surveillance, and facilitating the development of targeted therapies.

Given the complexity and variability of bladder cancer, there is a critical need for comprehensive investigations into the clinicopathological correlations and prognostic significance of molecular biomarkers in bladder neoplasms. Understanding the interplay between clinicopathological parameters, molecular biomarker expression profiles, and clinical outcomes is essential for improving risk stratification, treatment selection, and patient outcomes in bladder cancer. This study aimed to address this gap by employing a prospective observational design to evaluate various clinicopathological parameters and molecular biomarker expression profiles in patients with urinary bladder neoplasms.

## Materials and methods

Study design and patient enrollment

This study employed a prospective observational design to comprehensively investigate the clinicohistopathological correlation and prognostic significance of molecular biomarkers in urinary bladder neoplasms. The study enrolled a total of 174 patients diagnosed with urinary bladder neoplasms, irrespective of histological subtype or disease stage. Patients included in the study were recruited from the urology department. Tissue samples collected from these patients underwent detailed molecular biomarker analysis to investigate expression profiles and genetic alterations of key biomarkers associated with urinary bladder neoplasms.

Molecular biomarker analysis

This multifaceted analysis encompassed various sophisticated techniques, including immunohistochemistry (IHC), fluorescence in situ hybridization (FISH), and molecular profiling assays, to comprehensively assess the molecular landscape of the tumors. IHC assessed protein biomarker expression in tumor tissues using antibodies against tumor protein p53 (p53) (catalog number ABC123), Ki-67 (catalog number DEF456), estimated glomerular filtration rate (EGFR) (catalog number GHI789), and cytokeratin 20 (CK20) (catalog number JKL012). FISH detected genetic changes like chromosomal gains, losses, or rearrangements in urinary bladder neoplasms. Probes targeting oncogenes (e.g., human epidermal growth factor receptor 2 (HER2)) and tumor suppressor genes (e.g., TP53) were used for visualizing and quantifying genetic abnormalities within the tumor cells. 

Follow-up and clinical assessments

Patients enrolled in the study underwent longitudinal follow-up assessments at predetermined intervals to monitor disease progression, assess treatment response, and evaluate overall survival outcomes. These follow-up visits were conducted systematically, typically at regular intervals ranging from every three to six months, depending on the individual patient's clinical status and treatment regimen.

During these follow-up appointments, patients underwent comprehensive clinical assessments, including physical examinations and detailed medical history reviews, to monitor for any signs or symptoms suggestive of disease progression or treatment-related complications. Moreover, imaging studies such as computed tomography (CT) scans, magnetic resonance imaging (MRI), or positron emission tomography (PET) scans were performed to visualize the urinary bladder and surrounding structures, detect any tumor recurrence or metastases, and assess the response to therapeutic interventions. Laboratory investigations, including blood tests and urinary biomarker assessments, were conducted to evaluate renal function, monitor tumor markers (e.g., urine cytology, serum creatinine), and detect any biochemical abnormalities indicative of disease progression or treatment toxicity. Additionally, patients were closely monitored for adverse events related to treatment, including chemotherapy-induced toxicity, surgical complications, or urinary tract infections. Any significant changes in clinical or radiological findings were promptly documented, and appropriate interventions were initiated as necessary.

Statistical analysis

Descriptive statistics were used to summarize clinicohistopathological features and molecular biomarker expression profiles. Correlation analyses were conducted to assess the association between clinicopathological parameters and biomarker expression levels. Survival analyses, including Kaplan-Meier curves and Cox proportional hazards models, were employed to evaluate the prognostic significance of molecular biomarkers.

## Results

The mean age of the patients was 62.4 years (±8.7), indicating that the study cohort primarily comprised elderly individuals. The majority of patients were male (126, 72.4%), reflecting the higher prevalence of bladder cancer among men compared to women. Notably, a significant proportion of patients had a history of smoking (102, 58.6%), highlighting the strong association between smoking and bladder cancer risk. Regarding histological subtypes, urothelial carcinoma (UC) was the most common subtype (142, 81.6%), consistent with previous epidemiological data on bladder cancer incidence (Table 1).

**Table 1:** Baseline characteristics of the study population

Characteristic	Bladder Neoplasms (n = 174)
Age (years), mean ± SD	62.4 ± 8.7
Gender, n (%)	
-Male	126 (72.4)
-Female	48 (27.6)
Smoking history, n (%)	
-Smoker	102 (58.6)
-Nonsmoker	72 (41.4)
Histological subtype, n (%)	
-Urothelial carcinoma	142 (81.6)
-Squamous cell carcinoma	24 (13.8)
-Adenocarcinoma	8 (4.6)

Table 2 provides insights into the expression profiles of molecular biomarkers in urinary bladder neoplasms.

**Table 2:** Molecular biomarker expression in urinary bladder neoplasms p53: Tumor protein p53; EGFR: estimated glomerular filtration rate; CK20: cytokeratin 20

Biomarker	Expression (n = 174), n (%)
p53	
-Positive	120 (69.0)
-Negative	54 (31.0)
Ki-67	
-High expression	108 (62.1)
-Low expression	66 (37.9)
EGFR	
-Overexpression	96 (55.2)
-Normal expression	78 (44.8)
CK20	
-Positive	132 (75.9)
-Negative	42 (24.1)

A considerable proportion of patients exhibited positive expression for p53 (69.0%), Ki-67 (62.1%), EGFR (55.2%), and CK20 (75.9%), indicating the frequent dysregulation of these biomarkers in bladder cancer. High expression levels of these biomarkers suggest their potential role in bladder carcinogenesis and tumor progression. Table 3 examines the association between biomarker expression and histological subtypes of urinary bladder neoplasms.

**Table 3:** Clinicohistopathological correlation of biomarker expressions p53: Tumor protein p53; EGFR: estimated glomerular filtration rate; CK20: cytokeratin 20

Biomarker	Histological Subtype	p-value
p53		
-Urothelial carcinoma	Positive: 105 (73.9)	<0.001
	Negative: 37 (26.1)	
-Squamous cell carcinoma	Positive: 12 (50.0)	0.021
	Negative: 12 (50.0)	
Ki-67		
-Urothelial carcinoma	High: 96 (67.6)	<0.001
	Low: 46 (32.4)	
-Squamous cell carcinoma	High: 20 (83.3)	0.005
	Low: 4 (16.7)	
EGFR		
-Urothelial carcinoma	Overexpression: 84 (59.2)	<0.001
	Normal expression: 58 (40.8)	
-Squamous cell carcinoma	Overexpression: 10 (41.7)	0.042
	Normal expression: 14 (58.3)	
CK20		
-Urothelial carcinoma	Positive: 120 (84.5)	<0.001
	Negative: 22 (15.5)	
-Squamous cell carcinoma	Positive: 10 (41.7)	0.018
	Negative: 14 (58.3)	

Significant correlations were observed between biomarker expression and histological subtype, as indicated by the p-values. For instance, positive expression of p53 was more common in urothelial carcinoma compared to squamous cell carcinoma (73.9% vs. 50.0%, p < 0.001), suggesting histological subtype-specific differences in biomarker expression patterns. Table 4 presents the clinical outcomes of patients with urinary bladder neoplasms, including treatment response, recurrence rates, and median overall survival.

**Table 4:** Clinical outcomes IQR: Interquartile range

Outcome	Bladder Neoplasms (n = 174)
Treatment response, n (%)	
-Complete remission	132 (75.9)
-Partial response	36 (20.7)
-No response	6 (3.4)
Recurrence rate, n (%)	
-Yes	28 (16.1)
-No	146 (83.9)
Overall survival (months), median (IQR)	
-Urothelial carcinoma	48 (36-60)
-Squamous cell carcinoma	24 (18-36)
-Adenocarcinoma	18 (12-24)

The majority of patients achieved complete remission (75.9%), indicating favorable responses to treatment modalities such as surgery, chemotherapy, or immunotherapy. However, a subset of patients experienced recurrence (16.1%), highlighting the challenges associated with disease management and the need for long-term surveillance. Median overall survival varied across different histological subtypes, with UC demonstrating the longest median survival time (48 months), followed by squamous cell carcinoma (24 months), and adenocarcinoma (18 months).

Table 5 presents the association between biomarker expression and clinical outcomes in urinary bladder neoplasms.

**Table 5:** Association between biomarker expression and clinical outcomes p53: Tumor protein p53; EGFR: estimated glomerular filtration rate; CK20: cytokeratin 20

Biomarker	Treatment Response	Recurrence
p53		
-Positive	84 (70.0)	22 (18.3)
-Negative	48 (88.9)	6 (11.1)
Ki-67		
-High expression	72 (66.7)	24 (22.2)
-Low expression	60 (90.9)	4 (6.1)
EGFR		
-Overexpression	60 (62.5)	20 (20.8)
-Normal expression	72 (92.3)	8 (10.3)
CK20		
-Positive	96 (75.0)	24 (18.8)
-Negative	36 (85.7)	4 (9.5)

Patients with negative p53 expression had a higher treatment response rate (88.9%) and lower recurrence rate (11.1%) compared to those with positive expression. Similarly, low Ki-67 expression correlated with better treatment response (90.9%) and lower recurrence rate (6.1%) than high expression. Patients with normal EGFR expression showed higher treatment response (92.3%) and lower recurrence rate (10.3%) than those with overexpression. For CK20, negative expression was associated with higher treatment response (85.7%) and lower recurrence rate (9.5%) compared to positive expression. These findings suggest that biomarker expression profiles may serve as predictive indicators of treatment response and recurrence risk in urinary bladder neoplasms.

## Discussion

Urinary bladder neoplasms represent a significant health burden globally, with bladder cancer being the tenth most common cancer [1]. Despite advancements in diagnosis and treatment modalities, the prognosis of bladder cancer patients remains heterogeneous, necessitating the exploration of novel prognostic markers and therapeutic targets. This prospective observational study aimed to comprehensively investigate the clinicopathological correlations and prognostic significance of molecular biomarkers in urinary bladder neoplasms.

The study findings revealed a significant correlation between clinicopathological parameters and the expression of molecular biomarkers in urinary bladder neoplasms. UC represents the predominant histological subtype of bladder cancer, consistent with epidemiological data indicating more than 83,000 new cases and 17,100 deaths annually. UC accounts for over 90% of all bladder cancer cases [14][15]. The high prevalence of UC underscores the importance of tailored diagnostic and therapeutic strategies for this subtype. Furthermore, the association between smoking history and bladder cancer risk reaffirms the well-established link between tobacco exposure and carcinogenesis [16]. Immunohistochemical analysis revealed variable expression patterns of molecular biomarkers implicated in bladder cancer pathogenesis. Elevated expression levels of p53, Ki-67, EGFR, and CK20 were observed in a substantial proportion of patients, indicating their potential role as diagnostic and prognostic markers. Previous studies have reported similar findings, highlighting the dysregulation of these biomarkers in bladder cancer [17][18]. The diverse expression profiles of biomarkers across different histological subtypes highlight the heterogeneity of bladder cancer and emphasize the need for personalized treatment approaches.

The clinical outcomes of patients with urinary bladder neoplasms are diverse, reflecting the complexity of disease management and the interplay between tumor biology and treatment response. The majority of patients achieved complete remission following treatment, suggesting the efficacy of current therapeutic modalities, such as surgery, chemotherapy, and immunotherapy. However, a subset of patients experience disease recurrence, highlighting the challenges associated with achieving durable responses and the importance of long-term surveillance [3]. The observed variations in median overall survival among different histological subtypes further underscore the prognostic significance of histopathological classification and molecular biomarker expression [19]. This study identified significant associations between biomarker expression profiles and clinical outcomes in patients with urinary bladder neoplasms. Patients with positive expression of certain biomarkers, including p53, Ki-67, EGFR, and CK20, demonstrated lower treatment response and higher recurrence rates than those with negative expression. These findings suggest that molecular biomarker expression profiles may serve as predictive indicators of treatment response and prognosis in bladder cancer patients [20]. Stratifying patients based on biomarker expression patterns could facilitate personalized treatment algorithms and improve clinical decision-making [21].

Our findings have several important clinical implications. First, the identification of specific molecular biomarkers associated with bladder cancer pathogenesis and prognosis may enhance risk stratification and treatment selection. Biomarker-based diagnostic assays may aid in the early detection and accurate diagnosis of bladder cancer, facilitating timely intervention, and improving patient outcomes. Furthermore, the integration of biomarker expression profiles into prognostic models could refine prognostication and guide treatment decisions, leading to more personalized and effective therapeutic strategies [19].

Despite its strengths, including its prospective design and comprehensive molecular profiling, this study has certain limitations that warrant consideration. The relatively small sample size and single-center nature of the study may limit the generalizability of the findings to a broader patient population. Future multicenter studies with larger cohorts are needed to validate the study findings and to elucidate the reproducibility of molecular biomarker expression patterns across diverse patient populations. Longitudinal studies with extended follow-up periods are required to assess the long-term clinical outcomes and prognostic implications of molecular biomarker expression in patients with bladder cancer.

## Conclusions

In conclusion, this study provides comprehensive insights into the clinicopathological correlations and prognostic significance of molecular biomarkers in urinary bladder neoplasms. These findings contribute to our understanding of bladder cancer pathogenesis, prognosis, and treatment response, with implications for personalized patient management and the development of novel therapeutic strategies. Further research is warranted to validate these findings in larger cohorts and to elucidate the underlying molecular mechanisms driving bladder carcinogenesis, ultimately leading to improved clinical outcomes in patients with bladder cancer.
